# Patterns of Labour Interventions and Associated Maternal Biopsychosocial Factors in Australia: a Path Analysis

**DOI:** 10.1007/s43032-023-01219-7

**Published:** 2023-03-27

**Authors:** Habtamu Mellie Bizuayehu, Melissa L. Harris, Catherine Chojenta, Dominic Cavenagh, Peta M. Forder, Deborah Loxton

**Affiliations:** 1 School of Medicine and Public Health, College of Health, Medicine and Wellbeing, Centre for Women’s Health Research, The University of Newcastle, Newcastle, Australia; 2 Descriptive Epidemiology, Cancer Council Queensland, Brisbane, Australia; 3 Department of Public Health, Debre Markos University, Debre Markos, Ethiopia; 4 The First Nations Cancer & Wellbeing Research (FNCWR) Program, School of Public Health, The University of Queensland, Brisbane, Australia

**Keywords:** Episiotomy, Instrumental birth, Caesarean section birth, Labour interventions, Pregnancy outcomes, Longitudinal, Australia

## Abstract

In Australia, nearly half of births involve labour interventions. Prior research in this area has relied on cross-sectional and administrative health data and has not considered biopsychosocial factors. The current study examined direct and indirect associations between biopsychosocial factors and labour interventions using 19 years of population-based prospective data. The study included singleton babies among primiparous women of the 1973–1978 cohort of the Australian Longitudinal Study on Women’s Health. Data from 5459 women who started labour were analysed using path analysis. 42.2% of babies were born without intervention (episiotomy, instrumental, or caesarean delivery): Thirty-seven percent reported vaginal birth with episiotomy and instrumental birth interventions, 18% reported an unplanned caesarean section without episiotomy and/or instrumental interventions, and 3% reported unplanned caesarean section after episiotomy and/or instrumental interventions. Vaginal births with episiotomy and/or instrumental interventions were more likely among women with chronic hypertension (RRR(95%-CI):1.50(1.12–2.01)), a perceived length of labour of more than 36 h (RRR(95%-CI):1.86(1.45–2.39)), private health insurance (RRR(95%-CI):1.61(1.41–1.85)) and induced labour (RRR(95%-CI):1.69(1.46–1.94)). Risk factors of unplanned caesarean section without episiotomy and/or instrumental birth intervention included being overweight (RRR(95%-CI):1.30(1.07–1.58)) or obese prepregnancy (RRR(95%-CI):1.63(1.28–2.08)), aged ≥ 35 years (RRR(95%-CI):1.87(1.46–2.41)), having short stature (< 154 cm) (RRR(95%-CI):1.68(1.16–2.42)), a perceived length of labour of more than 36 h (RRR(95%-CI):3.26(2.50–4.24)), private health insurance (RRR(95%-CI):1.38(1.17–1.64)), and induced labour (RRR(95%-CI):2.56(2.16–3.05)). Prevention and management of hypertension, diabetes, and obesity during preconception and/or antenatal care are keys for reducing labour interventions and strengthening the evidence-base around delivery of best practice obstetric care.

**Supplementary Information:**

The online version contains supplementary material available at 10.1007/s43032-023-01219-7.

## Introduction

In Australia, almost half of births in 2021 required some form of assistance during labour, such as episiotomy (24%), use of instruments (e.g., vacuum extraction or forceps) (26.2%) [1], and/or unplanned caesarean Sect. 18.2%) [2]. This figure is more than double the 10–15% WHO recommended standard that seeks to improve newborn and maternal health [3–5]. In addition, the rates of labour interventions have relatively increased by about 10% over the past decade. For example, instrumental birth has increased by 12.7% [6]. Given that episiotomy, instrumental birth, and caesarean section are among the 12 core national maternity indicators used for monitoring maternal health care services quality [7], this signals a need for continuous measurement and identification of factors that may help to reduce these figures.

Past research has identified a range of maternal factors in relation to labour interventions, including biological factors (e.g., age, body mass index [BMI], height, history of miscarriage, diabetes, hypertension, asthma, length of labour, induction of labour) [2, 8–10], psychological factors (e.g., perceived stress, depression and anxiety) [11–13], and social factors (e.g., private health insurance, educational status, marital status, country of birth, maternal residential area) [2, 9, 10, 14, 15]. Most studies were based on secondary data from population-based birth registers and have not used a comprehensive model that considers the importance of each factor (biological, psychological and social). For example, some researchers did not adjust for possible confounding effects from psychological factors [9, 10, 14, 15], social factors [8, 11], and biological factors such as chronic diseases (hypertension, diabetes) [9, 13]. Further, most research has focused on direct associations between specific factors and labour interventions only [2, 8–12, 14–16]. However, indirect associations have been found [17], and the predictors of labour interventions are interrelated; for example, BMI is associated with diabetes and hypertension [18]; hypertension [19] is associated with an increased rate of induction of labour; area of residence is associated with diabetes and hypertension and with induction of labour [20].

Given that most prior research has used retrospective cross-sectional clinical data, has assessed direct associations between factors and labour interventions, and has not used a comprehensive theoretical framework, further research on the topic is warranted. To address this gap, this study used 19 years of nationally representative community-based prospective longitudinal data to describe patterns of labour interventions and maternal risk factors using the biopsychosocial framework [21]. Based on previous research, it was hypothesised that biological, psychological, and social factors would be associated directly or indirectly with labour interventions.

## Methods

### ALSWH and Participants

**Fig. 1:**
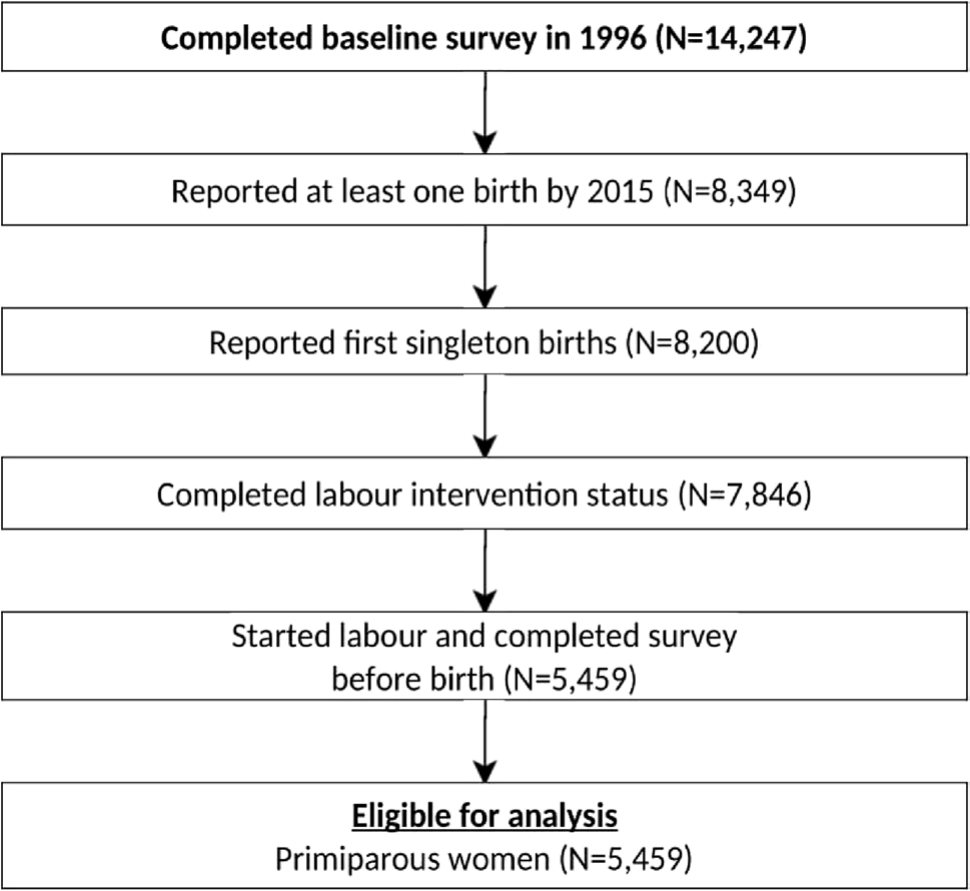
Selection process of eligible women from the ALSWH 1973-78 cohort for labour intervention, 1996-2015

Data for this study were drawn from the ALSWH which is a community-based cohort study among women in various age groups (born in 1973–1978, 1946–1951, and 1921–1926); the details of ALSWH are published and accessible elsewhere [22, 23]. Primiparous women in the 1973–1978 cohort of the ALSWH were eligible for this study if they (i) reported a singleton birth (≥ 20 weeks of gestation or ≥ 400 g of birth weight) during the observation period; (ii) had no missing data relating to the labour interventions examined; and (iii) completed the survey prior to the birth of their child. Using these criteria, 5459 women were included (Fig. 1).

### Measures

#### Outcome: Labour Interventions

For each birth, women were asked if they had experienced the following labour interventions: episiotomy (cutting of vagina/perineum); instrumental birth (forceps/vacuum); and caesarean section after labour started. In this study, the term labour intervention refers to episiotomy and/or instrumental birth and/or unplanned caesarean section interventions during childbirth. Labour intervention patterns were categorised into six groups: (i) spontaneous vaginal birth; (ii) vaginal birth assisted by episiotomy alone; (iii) vaginal birth assisted by instrumental birth alone; (iv) vaginal birth assisted by both episiotomy and instrumental birth; (v) unplanned caesarean section before episiotomy and/or instrumental birth interventions; (vi) unplanned caesarean birth after episiotomy and/or instrumental birth interventions.

#### Factors Associated with Labour Interventions

Maternal factors associated with labour interventions were grouped using a biopsychosocial approach [21], which recognises the potential impact of biological, psychological, and social factors. Women were surveyed seven times over a 19-year period (1996, 2000, 2003, 2006, 2009, 2012, and 2015) [23]. Unless specified, all variables presented below were captured in all surveys (Surveys 1–7).

#### Biological Factors

The included biological factors were women’s age (≤ 24 years, 25–34 years, and ≥ 35 years), prepregnancy BMI (< 18.5 kg/m^2^ [underweight], 18.5–24.9 kg/m^2^ [healthy weight], 25–29.9 kg/m^2^ [overweight] and (≥ 30 kg/m^2^ [obese]) [24], women’s height (< 154 cm [below 5% percentile] and ≥ 154 cm [5% percentile or above] [25], history of miscarriage, hypertension (chronic, gestational), diabetes (chronic, gestational), asthma, induction of labour, and perceived length of labour and preterm birth.

#### Psychological Risk Factors

Perceived stress was measured using the perceived stress questionnaire for young women [26] where the Likert scales ranged from ‘0’ (not at all stressed) to ‘4’ (extremely stressed). Mean stress scores were grouped as no-low stress (mean score ≤ 1) or moderate-high stress (mean score > 1). The perceived stress measurement has acceptable reliability (Cronbach’s alpha = 0.75), convergent validity with the ALSWH Life Events Check-list (*r* = 0.53), and is comparable with the commonly used 14-item perceived stress scale [27]. Antenatal depression and antenatal anxiety were considered affirmative if women reported diagnosis or treatment of these conditions during pregnancy.

#### Social Risk Factors

The social factors were educational status (no formal/school certificate, high school certificate, trade/certificate/diploma and degree/higher), relationship status (not partnered and partnered [married or de facto relationship] [28], partner violence (if reported ‘yes’ for the question “have you ever been in a violent relationship with a partner/spouse?”), private hospital insurance availability, country of birth (Australia or overseas), area of residence (major cities, inner regional, and outer-regional/remote, which were grouped using reported postcode and Accessibility/Remoteness Index of Australia Plus [ARIA +] measure).

### Statistical Analysis

**Fig. 2:**
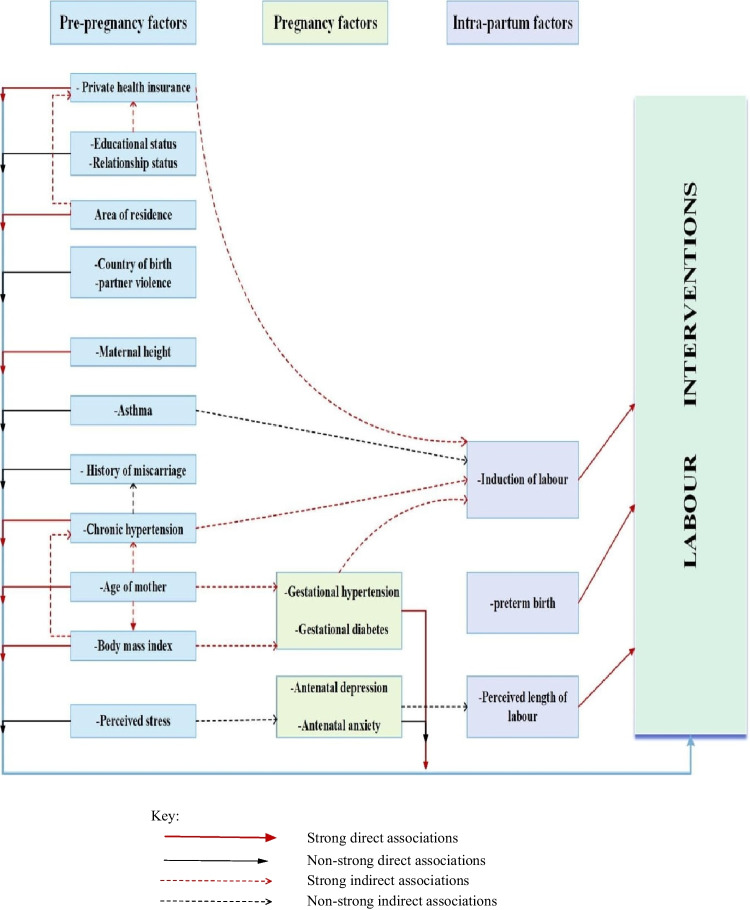
Path diagram for factors that have direct or indirect associations with labour interventions

The proportion of women who experienced each labour intervention pattern was described by dividing each category of the procedure patterns by the total number of births. Path analysis was used to examine the direct and indirect relationship of each predictor with labour intervention types [29]. A conceptual model was developed to understand the inter-relationship between maternal factors that may be associated directly or indirectly with labour interventions. The factors available in the dataset were classified as preconception, pregnancy, and intrapartum factors, and the relationship between these variables and labour intervention or among each other was developed based on the existing literature (e.g., [18–20]) and refined based on the research team’s discussions (Fig. 2). The final model was determined using a combination of clinical importance, Bayesian information criteria [30], and changes in effect size and corresponding confidence intervals of the effect size [31]. Variables with a strong association (indirect/direct) were retained in the final model. Both crude and adjusted relative risk ratios with corresponding 95% confidence intervals are presented.

All predictors had missing values below 6.6%, except for BMI (13.5%) and induction of labour (13.8%). Body mass index was imputed by participants’ self-mean BMI (i.e., calculated from all surveys responded by the participant [32]). After imputation, its missing value was reduced to 1%. A sensitivity analysis (including and excluding induction of labour in the model) was carried out due to their missing value being above 10% [32]. The analyses were carried out using Stata software (version 16).

## Results

### Characteristics of Participants

The characteristics of women who gave birth after labour started (*N* = 5459) are described in Table 1. Women’s mean age was 29.2 years (± 4.4 SD), with nearly three-quarters of them aged 25–34 years. Almost two-thirds (64%) of women had a healthy weight before pregnancy. Medical conditions were reported for up to one out of ten women, e.g., 8% for gestational hypertension. Most women had university qualifications (46%) and private health insurance (52%) (Table 1).

**Table 1:** Characteristics of Australian primiparous women in the ALSWH 1973–1978 cohort who had singleton births and reported starting of labour, 1996–2015

	Started labour (*N* = 5459)	
Characteristic	Label	*n* (%)
Preterm birth	No	4728 (92.6)
	Yes	376 (7.4)
	*Missing*	355
Low birth weight	No	5212 (95.5)
	Yes	247 (4.5)
High birth weight	No	4052 (85.6)
	Yes	684 (14.4)
	*Missing*	723
Induction of labour	No	2622 (55.7)
	Yes	2083 (44.3)
	*Missing*	754
Perceived length of labour > 36 h	No	4942 (90.6)
	Yes	514 (9.4)
	*Missing*	3
Age of mother (years)	≤ 24	1012 (18.5)
	25–34	3890 (71.3)
	≥ 35	557 (10.2)
BMI prior to pregnancy	Underweight	214 (4.5)
	Healthy weight	3038 (64.3)
	Overweight	999 (21.2)
	Obese	471 (10.0)
	*Missing*	737
Maternal height (cm)	< 154	250 (4.6)
	≥ 154	5157 (95.4)
	*Missing*	52
Miscarriage history	No	4782 (88.9)
	Yes	597 (11.1)
	*Missing*	80
Diabetes	No	5174 (94.8)
	Chronic	47 (0.9)
	Gestational	234 (4.3)
	*Missing*	4
Hypertension	No	4721 (86.5)
	Chronic	293 (5.4)
	Gestational	444 (8.1)
	*Missing*	1
Asthma	No	3926 (73.6)
	Yes	1406 (26.4)
	*Missing*	127
Perceived stress	None/minimal	3691 (67.8)
	Moderate/high	1749 (32.2)
	*Missing*	19
Emotional distress	No	3912 (71.7%)
	Yes	1192 (21.8%)
	*Missing*	355
Antenatal depression	No	4990 (97.8)
	Yes	112 (2.2)
	*Missing*	357
Antenatal anxiety disorder	No	4962 (97.3)
	Yes	136 (2.7)
	*Missing*	361
Education completed	No formal/school certificate	483 (9.0)
	High school certificate	1088 (20.2)
	Trade/certificate/diploma	1327 (24.7)
	Degree	2482 (46.1)
	*Missing*	79
Relationship status	Partnered	4299 (79.0)
	Non-partnered	1143 (21.0)
	*Missing*	17
Private hospital insurance	No	2620 (48.3)
	Yes	2803 (51.7)
	*Missing*	36
History of partner violence	No	4683 (85.9)
	Yes	767 (14.1)
	*Missing*	9
Area of residence	Major city	2962 (55.1)
	Inner regional	1446 (26.9)
	Outer regional/remote	967 (18.0)
	*Missing*	84
Country of birth	Overseas	366 (6.7)
	Australia	5066 (93.3)
	*Missing*	27

*BMI* Body mass index (classified according to WHO criteria)

### Labour Intervention Patterns

About two-fifths of women (42.2%) reported spontaneous vaginal birth, with the remaining births involving some sort of intervention. The rate of reported interventions was 16.3% for vaginal birth with both episiotomy and instrumental birth interventions, 10.5% for vaginal birth with episiotomy alone, 9.6% for vaginal birth with instrumental birth alone, 18.4% unplanned caesarean section before episiotomy and/or instrumental birth interventions, 2.8% for unplanned caesarean section after attempt of episiotomy and/or instrumental birth interventions.

**Fig. 3:**
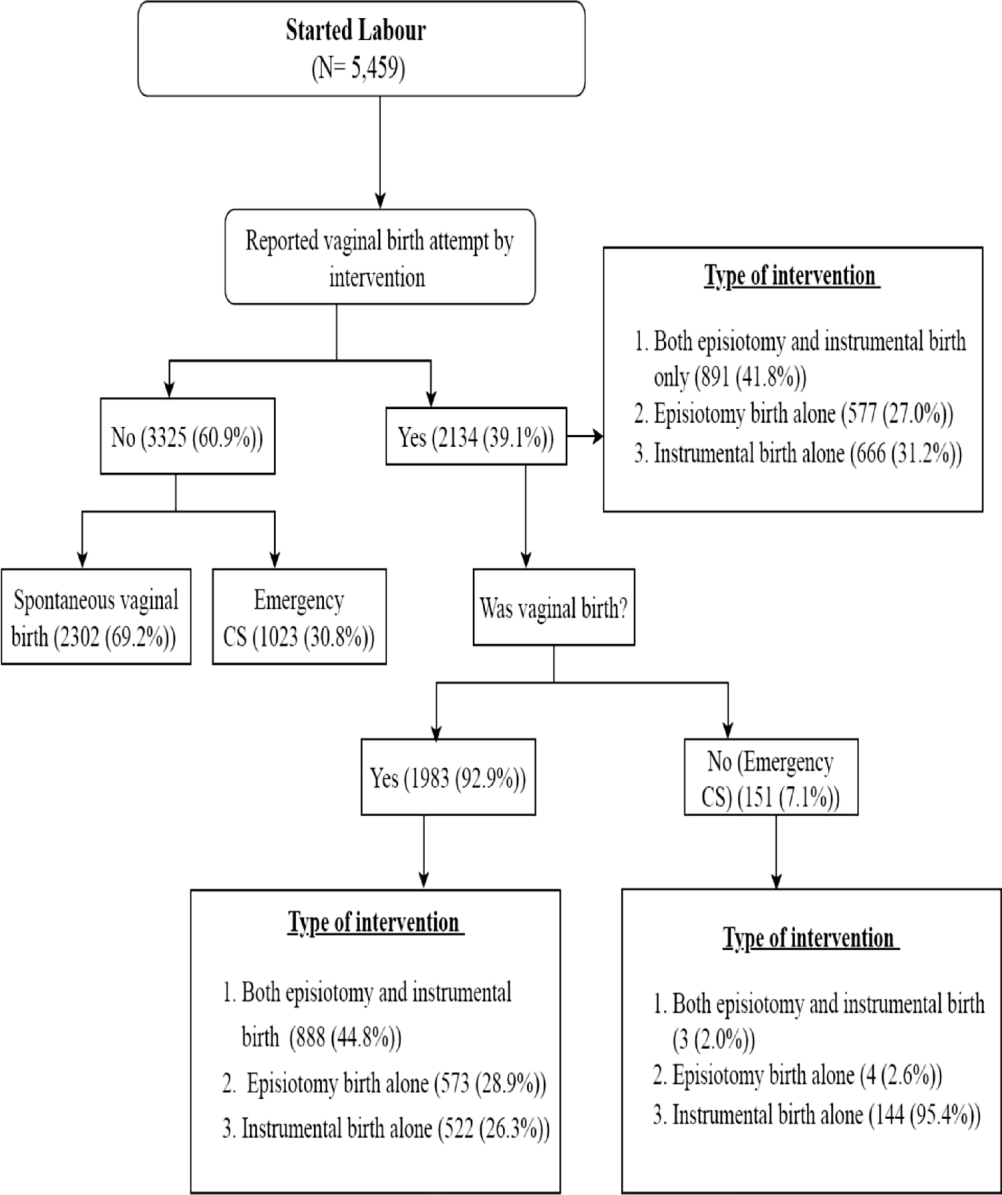
Labour intervention patterns of Australian primiparous women born in 1973-78 by reported labour onset for singleton newborns, 1996-2015

Two-fifths (39.1%) of women reported an episiotomy and/or an instrumental birth intervention. Episiotomy and instrumental birth were reported by 26.9% and 28.5% of women, respectively. Specific patterns of labour interventions are presented in Fig. 3. The pattern of labour interventions by women’s biopsychosocial characteristics are presented in Table 2.

**Table 2:** Rate of first singleton birth patterns across various characteristics of the ALSWH 1973–1978 cohort, 1996–2015

Variable	Label	Vaginal births					Unplanned caesarean section	
		Spontaneous births	Assisted birth^1^					
			Type of assisted births					
			Total assisted births	Both EI	Episiotomy alone	Instrumental alone	Before EI attempted ^1^	After EI attempted ^1^
		*N* = 2302	*N* = 1983	*N* = 888	*N* = 573	*N* = 522	*N* = 1023	*N* = 151
		*n* (%)	*n* (%)	*n* (%)	*n* (%)	*n* (%)	*n* (%)	*n* (%)
Preterm birth	No	1948 (91.0)	1759 (94.4)	800 (95.8)	494 (92.9)	465 (93.6)	898 (93.0)	123 (91.8)
	Yes	192 (9.0)	105 (5.6)	35 (4.2)	38 (7.1)	32 (6.4)	68 (7.0)	11 (8.2)
Low birth weight	No	2165 (94.0)	1923 (97.0)	866 (97.5)	554 (96.7)	503 (96.4)	978 (95.6)	146 (96.7)
	Yes	137 (6.0)	60 (3.0)	22 (2.5)	19 (3.3)	19 (3.6)	45 (4.4)	5 (3.3)
High birth weight	No	1758 (89.3)	1481 (85.7)	653 (83.8)	420 (86.4)	408 (87.9)	713 (77.9)	100 (81.3)
	Yes	211 (10.7)	248 (14.3)	126 (16.2)	66 (13.6)	56 (12.1)	202 (22.1)	23 (18.7)
Induction of labour	No	1273 (65.2)	906 (52.5)	389 (49.8)	277 (57.5)	240 (51.9)	374 (41.1)	69 (57.5)
	Yes	678 (34.8)	819 (47.5)	392 (50.2)	205 (42.5)	222 (48.1)	535 (58.9)	51 (42.5)
Perceived length of labour > 36 h	No	2171 (94.3)	1790 (90.3)	796 (89.7)	528 (92.1)	466 (89.3)	858 (84.0)	123 (81.5)
	Yes	131 (5.7)	192 (9.7)	91 (10.3)	45 (7.9)	56 (10.7)	163 (16.0)	28 (18.5)
Age of mother (years)	≤ 24	578 (25.1)	304 (15.3)	115 (13.0)	137 (23.9)	52 (10.0)	109 (10.7)	21 (13.9)
	25–34	1559 (67.7)	1479 (74.6)	683 (76.9)	389 (67.9)	407 (78.0)	748 (73.1)	104 (68.9)
	≥ 35	165 (7.2)	200 (10.1)	90 (10.1)	47 (8.2)	63 (12.1)	166 (16.2)	26 (17.2)
BMI prior to pregnancy	Underweight	101 (5.2)	86 (4.9)	37 (4.7)	29 (5.9)	20 (4.3)	21 (2.3)	6 (4.4)
	Healthy weight	1259 (65.3)	1169 (66.9)	514 (65.4)	355 (72.0)	300 (64.0)	537 (59.0)	73 (53.7)
	Overweight	393 (20.4)	351 (20.1)	170 (21.6)	81 (16.4)	100 (21.3)	216 (23.7)	39 (28.7)
	Obese	175 (9.1)	142 (8.1)	65 (8.3)	28 (5.7)	49 (10.4)	136 (14.9)	18 (13.2)
Maternal height (cm)	< 154	85 (3.7)	91 (4.6)	45 (5.1)	28 (5.0)	18 (3.5)	64 (6.3)	10 (6.7)
	≥ 154	2191 (96.3)	1875 (95.4)	839 (94.9)	537 (95.0)	499 (96.5)	952 (93.7)	139 (93.3)
Miscarriage history	No	2029 (88.9)	1752 (89.8)	782 (89.7)	519 (91.7)	451 (87.9)	873 (87.4)	128 (87.1)
	Yes	253 (11.1)	199 (10.2)	90 (10.3)	47 (8.3)	62 (12.1)	126 (12.6)	19 (12.9)
Diabetes	No	2190 (95.3)	1,893 (95.5)	842 (94.8)	558 (97.4)	493 (94.4)	956 (93.5)	135 (89.4)
	Yes	109 (4.7)	90 (4.5)	46 (5.2)	15 (2.6)	29 (5.6)	66 (6.5)	16 (10.6)
Hypertension	No	2014 (87.5)	1701 (85.8)	751 (84.6)	519 (90.6)	431 (82.6)	886 (86.6)	120 (79.5)
	Chronic	107 (4.7)	125 (6.3)	58 (6.5)	28 (4.9)	39 (7.5)	50 (4.9)	11 (7.3)
	Gestational	180 (7.8)	157 (7.9)	79 (8.9)	26 (4.5)	52 (10.0)	87 (8.5)	20 (13.2)
Asthma	No	1652 (73.3)	1418 (73.5)	625 (72.3)	416 (74.3)	377 (74.5)	752 (75.4)	104 (69.3)
	Yes	602 (26.7)	512 (26.5)	239 (27.7)	144 (25.7)	129 (25.5)	246 (24.6)	46 (30.7)
Perceived stress	None/minimal	1515 (66.0)	1387 (70.1)	620 (70.1)	400 (69.8)	367 (70.4)	691 (67.9)	98 (65.8)
	Moderate/high	780 (34.0)	592 (29.9)	265 (29.9)	173 (30.2)	154 (29.6)	326 (32.1)	51 (34.2)
Emotional distress	No	1870 (87.4)	1357 (72.8)	551 (66.0)	444 (83.6)	362 (72.8)	614 (63.5)	71 (53.0)
	Yes	270 (12.6)	506 (27.2)	284 (34.0)	87 (16.4)	135 (27.2)	353 (36.5)	63 (47.0)
Antenatal depression	No	2097 (98.0)	1818 (97.7)	816 (97.8)	517 (97.5)	485 (97.8)	945 (97.6)	130 (97.0)
	Yes	43 (2.0)	42 (2.3)	18 (2.2)	13 (2.5)	11 (2.2)	23 (2.4)	4 (3.0)
Antenatal anxiety	No	2092 (97.8)	1808 (97.2)	813 (97.5)	517 (97.5)	478 (96.4)	935 (96.7)	127 (95.5)
	Yes	46 (2.2)	52 (2.8)	21 (2.5)	13 (2.5)	18 (3.6)	32 (3.3)	6 (4.5)
Education completed	No formal/school certificate	235 (10.4)	158 (8.1)	60 (6.9)	56 (9.8)	42 (8.2)	75 (7.4)	15 (10.1)
	High school certificate	503 (22.2)	365 (18.7)	170 (19.5)	122 (21.4)	73 (14.3)	185 (18.3)	35 (23.6)
	Trade/certificate/diploma	591 (26.0)	454 (23.3)	200 (23.0)	121 (21.3)	133 (26.0)	246 (24.3)	36 (24.3)
	Degree	940 (41.4)	974 (49.9)	440 (50.6)	270 (47.5)	264 (51.6)	506 (50.0)	62 (41.9)
Relationship status	Partnered	1743 (75.9)	1605 (81.3)	733 (83.3)	448 (78.2)	424 (81.4)	828 (81.1)	123 (81.5)
	Non-partnered	553 (24.1)	369 (18.7)	147 (16.7)	125 (21.8)	97 (18.6)	193 (18.9)	28 (18.5)
Private hospital insurance	No	1282 (56.1)	829 (42.1)	355 (40.2)	245 (43.1)	229 (44.3)	438 (43.0)	71 (47.3)
	Yes	1003 (43.9)	1140 (57.9)	529 (59.8)	323 (56.9)	288 (55.7)	581 (57.0)	79 (52.7)
History of partner violence	No	1953 (85.0)	1732 (87.6)	780 (87.9)	495 (86.7)	457 (87.9)	875 (85.5)	123 (81.5)
	Yes	345 (15.0)	246 (12.4)	107 (12.1)	76 (13.3)	63 (12.1)	148 (14.5)	28 (18.5)
Area of residence	Major city	1199 (52.8)	1067 (54.7)	483 (55.5)	296 (52.6)	288 (55.7)	612 (61.0)	84 (55.6)
	Inner regional	641 (28.2)	520 (26.7)	233 (26.8)	150 (26.6)	137 (26.5)	242 (24.1)	43 (28.5)
	Outer regional/remote	430 (18.9)	363 (18.6)	154 (17.7)	117 (20.8)	92 (17.8)	150 (14.9)	24 (15.9)
Country of birth	Overseas	147 (6.4)	143 (7.3)	59 (6.7)	31 (5.5)	53 (10.2)	67 (6.6)	9 (6.0)
	Australia	2148 (93.6)	1827 (92.7)	824 (93.3)	536 (94.5)	467 (89.8)	951 (93.4)	140 (94.0)

*EI* Episiotomy and instrumental birth; *BMI* body mass index (classified according to the WHO criteria); Diabetes in this table is mainly for gestational form; chronic diabetes was reported by 47 women (vaginal birth without intervention [*n* = 15], vaginal birth with intervention [*n* = 14], unplanned caesarean section before episiotomy and/or instrumental birth intervention [*n* = 16], unplanned caesarean section after attempted episiotomy and/or instrumental birth intervention [*n* = 2])

^1^Episiotomy and/or instrumental birth intervention

### Factors Associated with Labour Interventions

The study showed that compared to spontaneous vaginal birth, vaginal birth with episiotomy and/or instrumental birth intervention was more likely among women with chronic hypertension than women without hypertension (1.5 times), women who reported > 36 h of perceived length of labour than ≤ 36 h (1.86 times), women with private health insurance than women without private health insurance (1.61 times) and for women who were induced compared to women who were not induced (1.69 times) (Table 3).

**Table 3:** Multivariate path analysis of final results of factors associated with labour intervention after labour started among primiparous women from the ALSWH 1973–1978 cohort, 1996–2015

Variable	Label	Vaginal birth		Unplanned caesarean section			
		With assisted birth^1^		Before assisted vaginal birth attempted^1^		After assisted vaginal birth attempted^1^	
		RRR^2^ (95% CI)	*p* value	RRR^2^ (95% CI)	*p* value	RRR^2^ (95% CI)	*p* value
Direct associations							
Age of mother (years)	≤ 24	0.62 (0.52–0.74)	< 0.001	0.40 (0.30–0.51)	< 0.001	0.57 (0.33–0.99)	0.05
	25–34	1					
	≥ 35	1.19 (0.94–1.50)	0.14	1.87 (1.46–2.41)	< 0.001	2.56 (1.57–4.16)	< 0.001
BMI prior to pregnancy	Under weight	0.99 (0.72–1.35)	0.95	0.54 (0.33–0.91)	0.02	0.65 (0.20–2.12)	0.48
	Healthy weight	1					
	Overweight	0.97 (0.82–1.14)	0.70	1.30 (1.07–1.58)	0.01	1.54 (1.02–2.32)	0.04
	Obese	0.78 (0.63–0.98)	0.03	1.63 (1.28–2.08)	< 0.001	1.24 (0.72–2.14)	0.43
Maternal height (cm)	< 154	1.39 (1.00–1.91)	0.05	1.68 (1.16–2.42)	0.01	2.16 (1.07–4.34)	0.03
	≥ 154	1					
Area of residence	Major city	1					
	Inner regional	1.10 (0.94–1.29)	0.22	0.87 (0.71–1.06)	0.16	1.17 (0.76–1.79)	0.47
	Outer regional/ remote	1.08 (0.90–1.28)	0.42	0.79 (0.63–0.99)	0.04	1.00 (0.61–1.65)	1.00
Hypertension	No	1					
	Chronic	1.50 (1.12–2.01)	0.01	0.99 (0.68–1.44)	0.95	1.69 (0.84–3.41)	0.14
	Gestational	1.17 (0.93–1.48)	0.19	1.11 (0.83–1.48)	0.47	2.01 (1.18–3.41)	0.01
Diabetes	No	1					
	Yes	0.85 (0.63–1.15)	0.29	1.09 (0.78–1.53)	0.60	1.83 (1.01–3.32)	0.05
Preterm birth	No	1					
	Yes	0.59 (0.45–0.75)	< 0.001	0.74 (0.55–1.00)	0.05	0.75 (0.39–1.47)	0.41
Perceived length of labour > 36 h	No	1					
	Yes	1.86 (1.45–2.39)	< 0.001	3.26 (2.50–4.24)	< 0.001	3.68 (2.24–6.03)	< 0.001
Private hospital insurance	No	1					
	Yes	1.61 (1.41–1.85)	< 0.001	1.38 (1.17–1.64)	< 0.001	1.36 (0.93–2.00)	0.12
Indirect associations							
		Underweight prior to pregnancy		Overweight prior to pregnancy		Obese prior to pregnancy	
Age of mother (years)	≤ 24	1.78 (1.31–2.42)	< 0.001	1.04 (0.87–1.24)	0.64	1.43 (1.16–1.76)	< 0.001
	25–34	1					
	≥ 35	0.62 (0.35–1.10)	0.10	1.14 (0.93–1.42)	0.21	1.11 (0.83–1.47)	0.48
		Chronic hypertension		Gestational hypertension			
Age of mother (years)	≤ 24	0.96 (0.70–1.32)	0.80	1.33 (1.05–1.69)	0.02		
	25–34	1					
	≥ 35	1.15 (0.80–1.67)	0.45	0.60 (0.40–0.89)	0.01		
BMI prior to pregnancy	Under weight	0.58 (0.23–1.42)	0.23	0.86 (0.47–1.56)	0.61		
	Healthy weight	1					
	Overweight	1.80 (1.34–2.42)	< 0.001	2.25 (1.79–2.83)	< 0.001		
	Obese	4.49 (3.35–6.02)	< 0.001	3.33 (2.56–4.34)	< 0.001		
		Diabetes					
Age of mother (years)	≤ 24	0.51 (0.34–0.78)	< 0.001				
	25–34	1					
	≥ 35	2.79 (2.07–3.75)	< 0.001				
BMI prior to pregnancy	Under weight	1.04 (0.50–2.17)	0.91				
	Healthy weight	1					
	Overweight	1.53 (1.13–2.07)	0.01				
	Obese	3.37 (2.48–4.58)	< 0.001				
Private hospital Insurance							
Education completed	No formal/school certificate	0.58 (0.45–0.74)	< 0.001				
	High school certificate	1					
	Trade/certificate/diploma	1.35 (1.14–1.60)	< 0.001				
	Degree/higher	3.17 (2.71–3.71)	< 0.001				
Relationship status	Partnered	1					
	Non–partnered	0.46 (0.40–0.53)	< 0.001				
Area of residence	Major city	1					
	Inner regional	0.51 (0.45–0.59)	< 0.001				
	Outer regional/remote	0.57 (0.49–0.67)	< 0.001				

*RRR* Relative risk ratio; *BMI* body mass index (classified according to the WHO criteria)

^1^Episiotomy and/or instrumental birth

^2^RRR for labour interventions were compared with spontaneous vaginal birth (reference), all variables in the path model were considered in the initial model, and the final model was determined based on the clinical importance, Bayesian information criterion, effect size, and corresponding confidence intervals of the effect size

Compared to spontaneous vaginal birth, the risk of unplanned caesarean section before episiotomy and/or instrumental birth interventions was higher among women aged ≥ 35 years than 25–34 years (1.87 times), women who were overweight prepregnancy compared to women who had a healthy weight (1.30 times), shorter stature women (< 154 cm) compared to taller women (≥ 154 cm) (1.68 times), women who reported > 36 h of perceived length of labour than ≤ 36 h (3.26 times), women with private health insurance than women who did not have a private health insurance (1.38 times) and for women who were induced than women who did not induced (2.56 times) (Table 3). Among women who gave birth with induction of labour compared to women who were not induced, there was a higher risk of vaginal birth with episiotomy/instrumental birth interventions (aRRR(95%-CI): 1.69(1.46–1.94)) or unplanned caesarean section before episiotomy and/or instrumental birth intervention (aRRR(95%-CI): 2.56(2.16–3.05)). Additional supplementary results are available online in Supplementary Table 1.

## Discussion

The hypothesis that biological, psychological, and social factors would be associated with labour interventions was partially supported. Analysis of nationally representative population-based longitudinal data revealed that biological factors (i.e., age, height, prepregnancy BMI, diabetes, hypertension, preterm birth, perceived length of labour, induction of labour) and social factors (i.e., area of residence, educational status, relationship status, private health insurance availability status) had either a direct or indirect association with labour interventions. However, associations between psychological factors (perceived stress, antenatal depression, and antenatal anxiety) and labour interventions were weak. Previous literature was largely based on service use and clinical cross-sectional data, assessed direct associations only and had not used a comprehensive theoretical framework (e.g., [8, 9, 11, 12, 15, 16]). Therefore, this study contributes robust information about predisposing maternal factors for labour interventions.

In this study, a high proportion of women reported a birth by episiotomy (26.8%), instrumental intervention (25.9%), and unplanned caesarean Sect. (21.2%). The study result was similar to the Australian national estimate in 2021 for instrumental birth (26.2%) [1] and with estimates from Germany (22.9%) [33] and the UK (19.1%) [10] for unplanned caesarean Sect. (21.2%). Compared to this study result (26.8%), the national episiotomy rate was slightly lower (24%), and this could be due to including women who gave birth by planned caesarean section (and were not eligible for episiotomy) in the denominator in the national estimate [1]. However, the current study result was more than double compared to the WHO recommended standard (10–15%) of the procedure [3–5]. Hence, this research demonstrates an over-practice of labour interventions, which has no confirmed benefit, exposes the mother and baby to potential adverse consequences of the procedure, and is associated with unnecessary economic costs [34]. Therefore, increasing women’s informed decision power by providing educational material regarding evidence on labour interventions including benefits and consequences and risk factors associated with the procedures, as well as enhancing further training for health professionals (including the updating of clinical guidelines), providing periodic auditing and feedback on rates of labour interventions could be important in the promotion of evidence-based practice of labour interventions [35].

Regarding the association between labour interventions and psychological factors (perceived stress, antenatal depression, and antenatal anxiety), there is little consensus among researchers. Some researchers have found strong associations between psychological factors and labour interventions [12, 13], while this study found weak evidence of both direct and indirect associations. This is supported by within the literature [36]. Possible reasons for such inconsistent evidence are likely attributed to variations in study design, measurement differences, sample size, study population, and health system variations in the support of women during pregnancy. This study was based on nationally representative cohort data with a large sample size, and a comprehensive set of factors was controlled for during analysis.

Modifiable risk factors associated with labour interventions in this study included chronic diseases (hypertension, diabetes) and being overweight/obese prepregnancy (with respect to unplanned caesarean section) [8, 16] and are comparable with past research. Importantly, the current study points to the importance of strengthening chronic diseases (such as diabetes and hypertension) prevention and management approach during the preconception and antenatal period in order to reduce the need for intervention during labour. Access to preconception care serves to prepare women to conceive during better periods of health to improve outcomes for both mother and child (including minimising the need for labour interventions). However, less than half of women with chronic diseases utilise preconception care (46.8%) [37] and/or contraception services for contraceptive counselling (48.8%), and two-fifths (40%) of pregnancies among women with chronic diseases are unplanned [38]. There is also limited awareness about preconception care among general practitioners who are the main providers of the service with only 53% having awareness of preconception care guidelines. They have also low motivation to provide the service [39, 40]. Hence, further strengthening service providers’ awareness and motivation, contraceptive counselling, and prevention of unplanned pregnancy among women with chronic diseases could far reaching impacts on birth outcomes including the prevention of labour interventions.

While antenatal care is another critical period for the management of chronic diseases, only 56% of women met Australian national antenatal care guideline recommendations for antenatal care initiation (starting antenatal care before 10 weeks of pregnancy). In addition, 6% did not attend five or more antenatal care visits during pregnancy [6]. Strengthening antenatal care provision could indirectly reduce labour interventions by early identification and management of hypertension and diabetes.

Compared to spontaneous vaginal birth, vaginal births assisted by episiotomy and/or instrumental birth were less likely among women who were obese prepregnancy, and this result is consistent with prior research [8]. This could be due to early unplanned caesarean section interventions before cervical dilatation (e.g., unplanned caesarean section was more likely among women who were overweight/obese prepregnancy in this study), which may be delayed among obese women because they have a slower progress of labour and lower uterine contractions [41]. Other possible reasons could be health professional’s perception of the difficulty in performing the procedure, skill limitations, and/or fear of a failed procedure [42]. As the proportion of obese women among childbearing women is increasing, providing evidence-based episiotomy and/or instrumental birth interventions and reducing unplanned caesarean section births could be necessary [42]. The study results highlight the importance of monitoring weight during the preconception and antenatal periods through exercise, individualised tailored nutritional counselling and education, and monitoring of weight gain in order to reduce unplanned caesarean section [43]. Given that cervical dilatation, health professional related factors (skill, attitude), and newborn-related factors (presentation, position) associated with episiotomy and/or instrumental birth interventions among women who were obese prepregnancy were not measured in this study; it is recommended that further research focus on this area.

### Strengths and Limitations

This study has strengths in terms of its data source and analysis method. Nationally representative population-based data were used. The direct and indirect associations between biopsychosocial factors (collected prospectively over 19 years) and labour interventions were assessed using path analysis. Most of the previous literature is limited to secondary cross-sectional data and direct associations of factors. This study, however, has a few limitations, including due to using self-report data (e.g., potential misclassification bias). Nevertheless, very good agreement between self-reported labour interventions with medical register reports has been found in the UK [44] and Norway [45]. For example, researchers in the UK found very good agreement between antenatal records and self-reported caesarean section (Kappa = 1.00) and self-reported instrumental birth (Kappa = 93.7%) [44]. Perinatal outcomes are highly socially valued data, repeatedly shared with family/friends, remembered frequently, and reported accurately [46]. In addition, a strong agreement between self-reported diabetes [47, 48], hypertension [47, 48], and asthma [48] and administrative medical records was found in Australia (ALSWH data) [47] and Canada [48]. Good agreement between self-reported and more objective measures was also found for other factors, including induction of labour [45], length of labour > 36 h [44], and depression and anxiety [49]. Furthermore, our estimate of labour interventions [6] and the direction and strength of factors associated with labour interventions were comparable with previous research [8, 9, 16, 36]. Therefore, the results may not be influenced to a large extent by possible misclassification bias.

## Conclusion

Both biological and social factors were directly and indirectly associated with labour interventions. Further strengthening of prevention, early identification, and management of chronic diseases (diabetes, hypertension) and monitoring weight using exercise and nutritional advice could help to reduce labour interventions. Promoting evidence-based labour interventions by increasing women’s informed decision-making power, curriculum modification, and on-the-job training to increase the skill of managing labour progress, monitoring, and evaluation of procedures is beneficial not only for reducing labour interventions and for promoting evidence-based obstetric care best practice but also for achieving optimal pregnancy outcomes [7, 35].

## Supplementary Information

Below is the link to the electronic supplementary material.

Supplementary file1 (DOCX 47 KB)

## Data Availability

Not applicable due to data privacy protocol.
