# Supplementary material for: Patterns of Labour Interventions and Associated Maternal Biopsychosocial Factors in Australia: a Path Analysis
Source: Reprod Sci. 2023 Mar 27;30(9):2767–79. doi: 10.1007/s43032-023-01219-7 (PMC10480095; doi:10.1007/s43032-023-01219-7)
Supplement: Supplementary file 1 — Supplementary file1 (DOCX 47 KB) [file 43032_2023_1219_MOESM1_ESM.docx]

Supplementary Table 1: A sensitivity analysis of induction of labour in the final path analysis results of factors associated with labour intervention after labour started among primiparous women from the ALSWH 1973-1978 cohort, 1996-2015.

|  |  | **Vaginal birth** | | | | **Unplanned caesarean section** | | | | | | | | |
| --- | --- | --- | --- | --- | --- | --- | --- | --- | --- | --- | --- | --- | --- | --- |
|  |  | **With assisted birth ^1^** | | | | **Before assisted birth attempted ^1^** | | | | **After assisted birth attempted ^1^** | | | | |
| **Variable** | Label | **aRRR^2^** | **95%CI** | | **p-value** | **aRRR^2^** | **95%CI** | | **p-value** | **aRRR^2^** | **95%CI** | | **p-value** |  |
|  | **Direct associations** | | | | | | | | | | | | | |
| **Age of mother (years)** | ≤24 | 0.58 | 0.48 | 0.71 | < 0.001 | 0.39 | 0.30 | 0.52 | < 0.001 | 0.56 | 0.31 | 1.03 | 0.06 |  |
|  | 25-34 | 1 |  |  |  |  |  |  |  |  |  |  |  |  |
|  | ≥35 | 1.17 | 0.92 | 1.48 | 0.19 | 1.84 | 1.42 | 2.37 | <0.001 | 2.64 | 1.61 | 4.32 | <0.001 |  |
| **BMI prior to pregnancy** | Under weight | 1.03 | 0.75 | 1.42 | 0.85 | 0.58 | 0.34 | 0.97 | 0.04 | 0.74 | 0.23 | 2.42 | 0.62 |  |
|  | Healthy weight | 1 |  |  |  |  |  |  |  |  |  |  |  |  |
|  | Overweight | 0.89 | 0.76 | 1.06 | 0.20 | 1.11 | 0.91 | 1.36 | 0.31 | 1.54 | 1.00 | 2.38 | 0.05 |  |
|  | Obese | 0.71 | 0.56 | 0.89 | <0.001 | 1.43 | 1.11 | 1.85 | 0.01 | 1.36 | 0.78 | 2.40 | 0.28 |  |
| **Maternal height (cm)** | <154 | 1.47 | 1.04 | 2.08 | 0.03 | 1.82 | 1.23 | 2.69 | <0.001 | 2.30 | 1.10 | 4.80 | 0.03 |  |
|  | ≥154 | 1 |  |  |  |  |  |  |  |  |  |  |  |  |
| **Area of residence** | Major city | 1 |  |  |  |  |  |  |  |  |  |  |  |  |
|  | Inner regional | 1.09 | 0.93 | 1.28 | 0.30 | 0.87 | 0.70 | 1.06 | 0.17 | 1.12 | 0.71 | 1.76 | 0.63 |  |
|  | Outer regional/remote | 1.06 | 0.88 | 1.27 | 0.57 | 0.80 | 0.63 | 1.02 | 0.07 | 1.07 | 0.64 | 1.80 | 0.79 |  |
| **Hypertension** | No | 1 |  |  |  |  |  |  |  |  |  |  |  |  |
|  | Chronic | 1.39 | 1.02 | 1.88 | 0.03 | 0.86 | 0.58 | 1.27 | 0.44 | 1.54 | 0.74 | 3.21 | 0.25 |  |
|  | Gestational | 0.99 | 0.77 | 1.27 | 0.91 | 0.90 | 0.67 | 1.22 | 0.50 | 1.77 | 0.99 | 3.17 | 0.06 |  |
| **Diabetes** | No | 1 |  |  |  |  |  |  |  |  |  |  |  |  |
|  | Yes | 0.85 | 0.62 | 1.16 | 0.30 | 1.02 | 0.72 | 1.44 | 0.93 | 1.65 | 0.87 | 3.13 | 0.12 |  |
| **Preterm birth** | No | 1 |  |  |  |  |  |  |  |  |  |  |  |  |
|  | Yes | 0.60 | 0.45 | 0.78 | <0.001 | 0.82 | 0.59 | 1.13 | 0.22 | 0.79 | 0.39 | 1.60 | 0.52 |  |
| **Perceived length of labour >36 hours** | No | 1 |  |  |  |  |  |  |  |  |  |  |  |  |
|  | Yes | 1.79 | 1.38 | 2.31 | <0.001 | 3.05 | 2.31 | 4.01 | <0.001 | 2.92 | 1.69 | 5.03 | <0.001 |  |
| **Private hospital insurance** | No | 1 |  |  |  |  |  |  |  |  |  |  |  |  |
|  | Yes | 1.54 | 1.34 | 1.78 | <0.001 | 1.34 | 1.12 | 1.60 | <0.001 | 1.34 | 0.90 | 2.01 | 0.15 |  |
| **Induction of labour** | No | 1 |  |  |  |  |  |  |  |  |  |  |  |  |
|  | Yes | 1.69 | 1.46 | 1.94 | <0.001 | 2.56 | 2.16 | 3.05 | <0.001 | 1.17 | 0.79 | 1.73 | 0.44 |  |
| **Indirect associations** | | | | | | | | | | | | | | |
|  |  | **Underweight prior to pregnancy** | | | | **Overweight prior to pregnancy** | | | | **Obese prior to pregnancy** | | | | |
| **Age of mother (years)** | ≤24 | 1.78 | 1.31 | 2.42 | <0.001 | 1.04 | 0.87 | 1.24 | 0.64 | 1.43 | 1.16 | 1.76 | <0.001 |  |
|  | 25-34 | 1 |  |  |  |  |  |  |  |  |  |  |  |  |
|  | ≥35 | 0.62 | 0.35 | 1.10 | 0.10 | 1.14 | 0.93 | 1.42 | 0.21 | 1.11 | 0.83 | 1.47 | 0.48 |  |
|  |  | **Chronic hypertension** | | | | **Gestational hypertension** | | | |  |  |  |  | |
| **Age of mother (years)** | ≤24 | 0.96 | 0.70 | 1.32 | 0.80 | 1.33 | 1.05 | 1.69 | 0.02 |  |  |  |  |  |
|  | 25-34 | 1 |  |  |  |  |  |  |  |  |  |  |  |  |
|  | ≥35 | 1.15 | 0.80 | 1.67 | 0.45 | 0.60 | 0.40 | 0.89 | 0.01 |  |  |  |  |  |
| **BMI prior to pregnancy** | Under weight | 0.58 | 0.23 | 1.42 | 0.23 | 0.86 | 0.47 | 1.56 | 0.61 |  |  |  |  |  |
|  | Healthy weight | 1 |  |  |  |  |  |  |  |  |  |  |  |  |
|  | Overweight | 1.80 | 1.34 | 2.42 | <0.001 | 2.25 | 1.79 | 2.83 | <0.001 |  |  |  |  |  |
|  | Obese | 4.49 | 3.35 | 6.02 | <0.001 | 3.33 | 2.56 | 4.34 | <0.001 |  |  |  |  |  |
|  |  | **Diabetes** | | |  |  |  |  |  |  |  |  |  |  |
| **Age of mother (years)** | ≤24 | 0.51 | 0.34 | 0.78 | <0.001 |  |  |  |  |  |  |  |  |  |
|  | 25-34 | 1 |  |  |  |  |  |  |  |  |  |  |  |  |
|  | ≥35 | 2.79 | 2.07 | 3.75 | <0.001 |  |  |  |  |  |  |  |  |  |
| **BMI prior to pregnancy** | Under weight | 1.04 | 0.50 | 2.17 | 0.91 |  |  |  |  |  |  |  |  |  |
|  | Healthy weight | 1 |  |  |  |  |  |  |  |  |  |  |  |  |
|  | Overweight | 1.53 | 1.13 | 2.07 | 0.01 |  |  |  |  |  |  |  |  |  |
|  | Obese | 3.37 | 2.48 | 4.58 | <0.001 |  |  |  |  |  |  |  |  |  |
|  |  | **Private hospital insurance** | | | | | | | |  |  |  |  | |
| **Education completed** | No formal/school certificate | 0.58 | 0.45 | 0.74 | <0.001 |  |  |  |  |  |  |  |  |  |
|  | High school certificate | 1 |  |  |  |  |  |  |  |  |  |  |  |  |
|  | Trade/certificate/diploma | 1.35 | 1.14 | 1.60 | <0.001 |  |  |  |  |  |  |  |  |  |
|  | Degree | 3.17 | 2.71 | 3.71 | <0.001 |  |  |  |  |  |  |  |  |  |
| **Relationship status** | Partnered | 1 |  |  |  |  |  |  |  |  |  |  |  |  |
|  | Non-partnered | 0.46 | 0.40 | 0.53 | <0.001 |  |  |  |  |  |  |  |  |  |
| **Area of residence** | Major city | 1 |  |  |  |  |  |  |  |  |  |  |  |  |
|  | Inner regional | 0.51 | 0.45 | 0.59 | <0.001 |  |  |  |  |  |  |  |  |  |
|  | Outer regional/remote | 0.57 | 0.49 | 0.67 | <0.001 |  |  |  |  |  |  |  |  |  |
|  |  | **Induction of labour** | | | |  |  |  |  |  |  |  |  |  |
| **Hypertension** | No | 1 |  |  |  |  |  |  |  |  |  |  |  |  |
|  | Chronic | 1.60 | 1.23 | 2.07 | <0.001 |  |  |  |  |  |  |  |  |  |
|  | Gestational | 3.85 | 3.06 | 4.85 | <0.001 |  |  |  |  |  |  |  |  |  |
| **Diabetes** | No | 1 |  |  |  |  |  |  |  |  |  |  |  |  |
|  | Yes | 1.64 | 1.27 | 2.13 | <0.001 |  |  |  |  |  |  |  |  |  |
| **Private hospital insurance** | No | 1 |  |  |  |  |  |  |  |  |  |  |  |  |
|  | Yes | 1.22 | 1.08 | 1.37 | <0.001 |  |  |  |  |  |  |  |  |  |

Notes: RRR = relative risk ratio; BMI= Body Mass Index (classified according to the WHO criteria).

^1^ Episiotomy and/or instrumental birth

**^2^** RRR for labour interventions were compared with spontaneous vaginal birth (reference), all variables in the path model were considered in the initial model and the final model was determined based on the clinical importance, Bayesian information criterion, effect size and corresponding confidence intervals of the effect size.
